# Environmental triggers of autoimmune hepatitis: a clinical perspective from Yemeni patients

**DOI:** 10.3389/fimmu.2026.1757477

**Published:** 2026-03-10

**Authors:** Waleed Qassem Farie, Saleh Salem Bahaj, Anwar Kassem Al-Madhagi, Sarosh Sher Ali, Aref Noman, Waleed Yahya Alkassar

**Affiliations:** 1 Department of Microbiology and Immunology, Faculty of Medicine and Health Sciences, Sana’a University, Sana’a, Yemen; 2 Department of Pediatric and Child Health, Aga Khan University, Karachi, Pakistan

**Keywords:** autoimmune hepatitis, environmental exposure, Khat chewing, pesticide exposure, viral hepatitis, Yemen, vitamin D deficiency

## Abstract

**Introduction:**

Autoimmune hepatitis is a chronic inflammatory liver disease in which the immune system attacks liver tissue. While genetic predisposition plays a role, environmental factors are increasingly recognized as contributors to disease onset and progression. This study aimed to examine the association between environmental exposures and autoimmune hepatitis in a Yemeni population.

**Methodology:**

A case-control study was conducted, including 93 patients with clinically diagnosed autoimmune hepatitis and 280 age- and sex-matched healthy controls. Data was collected through structured interviews and laboratory analyses. Environmental exposures assessed included residence in different temperature zones, history of viral infections, medication use, pesticide exposure, and the habit of chewing Khat. Liver biopsy findings and vitamin D levels were evaluated to assess disease severity. Statistical comparisons were performed using odds ratios and confidence intervals to determine associations.

**Results:**

Khat chewing was significantly more common in autoimmune hepatitis patients than in controls (65.6% versus 42.1%, OR: 2.6; 95% CI: 1.6–4.3, p < 0.001). Exposure to medications known to induce autoimmune reactions, such as nitrofurantoin and minocycline, was also higher among patients (27.9% versus 1.8%, OR: 21.3; 95% CI: 7.9–57.7, p < 0.001). Living in warm temperature zones and exposure to pesticides (OR: 13.1; 95% CI: 2.7–62.8, p < 0.001) were both significantly associated with increased disease risk. Patients with these exposures also demonstrated higher liver enzyme levels and more advanced fibrosis on biopsy. Vitamin D deficiency was associated with greater disease severity.

**Conclusions:**

These findings highlight the important role of environmental factors, particularly Khat chewing and pesticide exposure, in the development and progression of autoimmune hepatitis in Yemen. Public health interventions addressing these exposures may help reduce disease burden.

## 1 Introduction

Autoimmune Hepatitis (AIH) is a chronic liver disorder in which the body’s immune system targets and destroys the liver cells, leading to inflammation, fibrosis, and, if left untreated, it leads to cirrhosis and liver failure (1). The pathogenesis of AIH includes both genetic predisposition and environmental factors as contributing factors to the disease onset and its progression (2). According to research, specific human leukocyte antigen (HLA) alleles have been linked as genetic factors. Similarly, infections, medications, toxins, and dietary habits have been recognized as key contributors to the development and exacerbation of AIH (3).

The role of environmental triggers in autoimmune diseases, including AIH, is significant as they can interact with a genetically predisposed immune system, thus initiating an autoimmune response (4). Viral infections like Hepatitis A, B, and C are well documented as environmental triggers of AIH (5, 6). Furthermore, the use of drugs such as nitrofurantoin and minocycline, and environmental toxins like pesticides and solvents, has been linked to AIH exacerbations in various populations (7, 8).

The literature mostly focuses on AIH in Western and Asian populations; less is known about the environmental factors influencing AIH in Middle Eastern countries, like Yemen. Yemen has unique challenges due to its distinct environmental conditions, dietary patterns, and healthcare infrastructure, all of which makes it an important region to study further in context of autoimmune diseases (9) Clinical reports suggest that environmental factors such as viral infections, malnutrition, and exposure to pollutants play a role in pathogenesis of AIH in Yemeni patients (10).

The objective of this study is to explore the environmental triggers of AIH from a clinical perspective in Yemeni patients, focusing on interactions between environmental exposures by examining clinical data from Yemeni cohorts, this study aims to contribute to a better understanding of how regional environmental conditions may influence the onset and progression of AIH, filling an important gap in the literature.

### 1.1 Research question

What environmental factors are associated with the development and severity of autoimmune hepatitis among Yemeni patients?

## 2 Methodology

### 2.1 Study design, setting, and population

This case-control study was conducted to investigate the environmental triggers of autoimmune hepatitis (AIH), with a particular focus on the potential roles of medications, viral infections, toxins, and lifestyle factors within a Yemeni cohort. AIH cases were recruited from the Department of Hepatology at the University of Science and Technology Hospital, Sana’a, a tertiary referral center serving multiple northern governorates in Yemen. The study included prevalent cases diagnosed between January 2022 and December 2024, using the International Autoimmune Hepatitis Group (IAHG) criteria. Exposure history was collected through structured interviews, focusing on environmental and lifestyle exposures occurring within the five years prior to symptom onset or diagnosis. Among 112 eligible AIH patients, 93 consented to participate (participation rate: 83%).

### 2.2 Eligibility criteria

#### 2.2.1 For AIH patients (cases)

The diagnosis of an AIH case was based on the Simplified IAIHG criteria (2008), which support the diagnosis of idiopathic AIH but do not exclude drug-induced liver injury (DILI). Patients with significant hepatotoxic drug exposure were further evaluated clinically for persistence of liver injury despite drug discontinuation, relapses, and need for prolonged immunosuppressive therapy. Exclusion criteria included the presence of acute viral hepatitis (HAV, HBV, and HCV), drug-induced liver injury, metabolic liver disease, or overlap syndromes with other autoimmune liver diseases.

#### 2.2.2 For healthy controls

Controls were recruited from general outpatient and family medicine clinics at the University of Science and Technology Hospital, Sana’a. These individuals were seeking care for non-chronic, non-autoimmune conditions (e.g., minor infections, musculoskeletal pain, routine health exams). Controls were selected from the same hospital catchment population as cases to ensure comparability and minimize selection bias and based on the clinical examination by specialist physicians, all selected controls were with no well-defined clinically and laboratory findings belong to AIH or other AIDs or chronic liver diseases.

Eligibility was determined through medical record review and structured interviews to exclude those with a history of autoimmune diseases, liver disorders, or positive serology for viral hepatitis. Controls were matched to cases by age (± 5 years) and sex. Of 310 individuals approached, 280 consented to participate, corresponding to a 90% participation rate.

### 2.3 Sample size

The sample size for this study was determined using Epi Info version 7. The calculation was based on a 95% confidence level and 80% statistical power, with a control-to-case ratio of 3:1. It was estimated that 4.9% of the cases would be exposed to the factor of interest (28). Based on these parameters, the required sample size was calculated to include 93 cases and 280 controls, resulting in a total sample size of 373 participants.

### 2.4 Assessment

To assess the environmental triggers of AIH, we utilized patient history forms and structured interviews. The environmental factors considered in this study include temperature zones (warm, moderate, and cold regions), Viral infections (hepatitis B (HBV), hepatitis C (HCV), hepatitis A (HAV), and Epstein-Barr Virus (EBV)), and Medication history, focusing on drugs known to trigger AIH, like nitrofurantoin, minocycline, and statins. Toxin exposure included pesticide and industrial exposure details, and dietary and lifestyle factors, particularly focused on Kat chewing as a prevalent local habit.

Participants were asked to provide details about their exposures through the patient history forms. Specifically, data on Khat chewing habits, occupational exposure to pesticides, and history of viral infections were collected to assess their role as environmental factors potentially contributing to AIH development. Pesticide exposure was defined as self-reported direct contact with agricultural or pest control chemicals either at work or during home use. Structured interviews revealed that the most frequently reported pesticides included organophosphates (malathion, chlorpyrifos) and pyrethroids (permethrin, deltamethrin). Exposure was predominantly occupational and occurred during the five years preceding symptom onset. Use of personal protective equipment (PPE) was rare, and most individuals reported frequent unprotected handling of these chemicals. Khat exposure was assessed through structured interviews, including frequency, duration, and timing of use. “Regular use” was defined as daily chewing for ≥4 hours per day. The majority of AIH patients who used Khat reported starting this habit several years before symptom onset, with no participants initiating Khat use after diagnosis.

To minimize recall bias, interviews were conducted using standardized pilot-tested questionnaires administered by trained personnel. Participants were specifically asked to report Khat chewing and medication use occurring within the five years preceding symptom onset. Emphasis was placed on routine behaviors (e.g., daily chewing) and recent drug exposures, which are more reliably recalled.

Serological tests were used to confirm the diagnosis of AIH and assess the immune response in both cases and controls. The presence of autoantibodies like Anti-nuclear antibody (ANA), anti-smooth muscle antibodies (ASMA), anti-liver kidney microsomal antibodies (anti-LKM1), and anti-soluble liver antigen (anti-SLA/LP), which is specific to AIH, is indicative of autoimmune liver disease. The presence of autoantibodies can be supportive of AIH diagnosis in most patients.

In addition, liver enzyme levels (ALT, AST, and ALP) were measured as markers of liver function and inflammation. Viral hepatitis screening is a part of the diagnostic work-up of AIH and is conducted to exclude other causes of liver inflammation through serological screening. Hepatitis B surface antigen (HBsAg), anti-HBc IgG, anti-HCV antibody test, and anti-HAV IgG were performed to assess the potential triggering effect of past viral hepatitis contributing to AIH development.

Additionally, liver biopsies were obtained from AIH patients as part of the diagnostic work-up and analyzed for periportal inflammation, fibrosis, and circulatory changes. The biopsies were used to categorize the severity of AIH and correlate it with reported environmental exposures. The liver histological features were graded based on the necro inflammatory activity using the Ishak’s grading system.

### 2.5 Ethical considerations

The study was conducted in accordance with the Declaration of Helsinki. Ethical approval for the study was obtained from the Ethics Committee of the Faculty of Medicine and Health Sciences, Sana’a University, Republic of Yemen, on 22 October 2025, with file No.1792. Written informed consent was obtained from all participants before sample collection, adhering to the Declaration of Helsinki guidelines.

## 3 Statistical analysis

**Figure 1:**
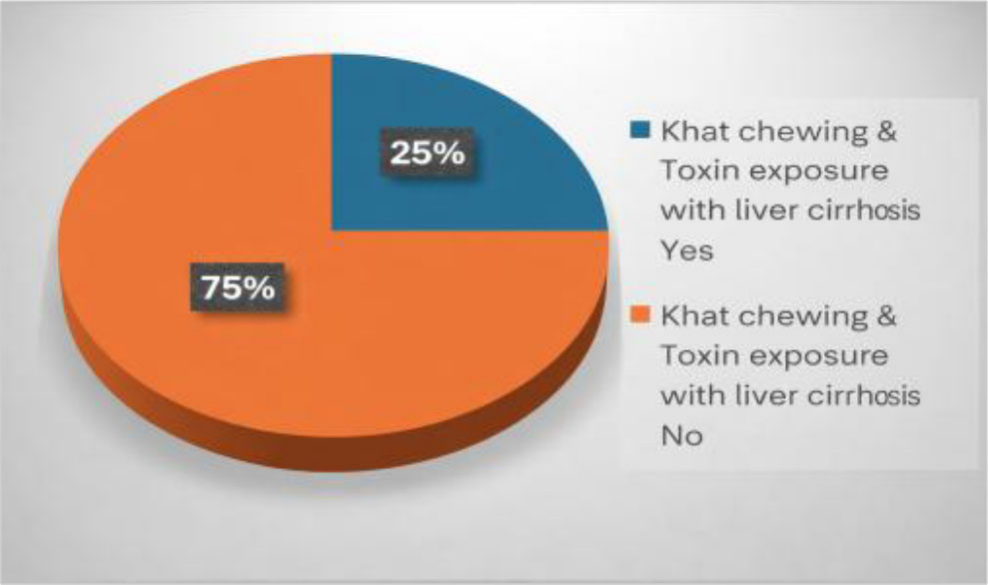
Khat chewing & toxin exposure with liver cirrhosis.

The demographic and clinical data were analyzed using SPSS version 25.0 (IBM Corp., Armonk, NY, USA). Differences in environmental exposures (such as viral infections, Khat chewing, and toxin exposure) between AIH patients and controls were analyzed using Pearson’s chi-square test or Fisher’s exact test for small sample sizes. The association between environmental factors (e.g., Khat chewing, viral infections) and AIH severity was assessed using odds ratios (ORs) and 95% confidence intervals (CIs). Continuous variables, such as liver enzyme levels (ALT, AST) and vitamin D levels, were compared between groups using Mann-Whitney U tests or one-way ANOVA, depending on data distribution. The median and interquartile range (IR) of liver enzymes in Khat chewers and those individuals with pesticides exposure were assessed. Statistical significance was set at p < 0.05 (**Figure 1**).

A multivariate logistic regression model was developed to adjust for potential confounders, including age, gender, occupation, and geographic zone. Adjusted odds ratios (aORs) and 95% confidence intervals were calculated to estimate independent associations between exposures and autoimmune hepatitis (**Table 1**).

**Table 1:** Characterization of the variables.

Variable	AIH N=93 (%)	Healthy control N=280 (%)
Gender		
Male	67 (72%)	187 (66.8%)
Female	26 (28%)	93 (33.2%)
Zone temperature		
Warm	46 (49.5%)	57 (20.4%)
Moderate	40 (43%)	154 (55%)
Cold	07 (7.5%)	69 (24.6%)
Khat chewing		
Yes	61 (65.6%)	118 (42.1%)
No	32 (34.4%)	162 (57.9%)
Nitrofurantoin and minocycline		
Yes	26 (27.9%)	5 (1.8%)
No	67 (62.1%)	275 (98.2%)
Toxin exposure		
Yes	8 (8.6%)	2 (0.7%)
No	85 (91.4%)	278 (99.3%)
Age (years) Mean ± SD	34.0 ± 10.4	32.7 ± 10.8

After adjusting OR for age, gender, and residence, Khat chewing, and pesticide exposure: Khat chewing (AOR = 2.601, 95% CI: 1.507–4.489, p = 0.001), residence in warm zones (AOR = 6.583, 95% CI: 2.598-16.677, p = 0.000) or moderate zones (AOR = 2.789, 95% CI: 1.127- 6.903, p = 0.027) and prior pesticide exposure (AOR = 13.799, 95% CI: 4.885–38.980, p = 0.000) remained significantly associated with AIH risk and those ORs >1.0 were suggest a susceptible effect The references were the residency in cold zones and non-exposed to Khat and pesticides.

## 4 Results

A total of 373 participants were included in the study, consisting of 93 AIH patients as cases and 280 healthy controls. The demographic characteristics of the participants showed no significant differences in terms of age and gender between the AIH group and the control group. The mean age of AIH patients was 34.0 ± 10.4 years, and the majority of them were male (72.0%), like the control group, which also had a mean age of 32.7 ± 10.8 years (**Table 2**).

**Table 2:** Logistic regression analysis for AIH risk factors.

Variables in the equation	χ^2^	P value	OR	95% CI for Exp	
				Lower	Upper
Cold Temperature Zone	18.606	0.000			
Warm Zone Temperature	15.785	0.000	6.583	2.598	16.677
Moderate Zone Temperature	4.918	0.027	2.789	1.127	6.903
Chewing Khat	11.776	0.001	2.601	1.507	4.489
Pesticide exposure	24.541	0.000	13.799	4.885	38.980

The medication history of AIH patients revealed that 27.9% had used medications that are known to trigger autoimmune hepatitis, such as nitrofurantoin and minocycline, compared to just 1.8% in the control group (OR: 21.3, 95% CI: 7.9-57.7, p-value <0.001). Toxin exposure was found to be more common in patients with AIH, with 8.6% reporting occupational pesticide exposure, a significantly higher rate compared to 0.7% in the control group (OR: 13.1, 95% CI: 2.7-62.8, p-value <0.001).

Although not strictly an environmental trigger, liver biopsy results also showed the presence of peri-portal inflammation and moderate to severe fibrosis in the majority of AIH patients, with 86% showing liver injury. Environmental triggers like Khat chewing and toxin exposure were associated with more severe histological findings. Liver cirrhosis was observed in 12.9% of AIH patients, with a rate of cirrhosis (25.0%) among those exposed to Khat and pesticides.

Statistical analysis confirmed strong associations between environmental exposures and AIH severity. Khat chewing and pesticide exposure were associated with higher mean rank liver enzyme levels. The median and interquartile range (IQR) of liver enzymes in this two groups alongside the mean rank were provided a clear clinical picture of the liver enzymes increasing.

Vitamin D deficiency was also found to be linked with more severe AIH (p-value <0.001), as patients with lower vitamin D levels have higher rates of liver cirrhosis according to Child-Pugh score. This finding supports the suggestive role of vitamin D as a prognostic biomarker in AIH patients with no clear mechanism. Vitamin D is not a primary cause of AIH, but vitamin D may be promotes immune tolerance, and it is deficiency may contribute to immune dysregulation or enhance non-response or poor response to standard therapy.

The significant elevation of IgG and the presence of serum autoantibodies (ANA, ASMA) in 93.6% of our patients are strongly favor idiopathic AIH-1 with completely absence of AIH-2 (**Table 3**).

**Table 3:** The mean rank of liver enzymes in association with Khat chewing and pesticide exposure in AIH patients.

Mean rank	Khat chewing		Mann-Whitney U test	P-value
	Yes	No		
ALT	209.2	166.5	13385.5	0.001
AST	201.2	173.9	14813.5	0.01
ALP	200.8	174.2	14886.5	0.02
Mean rank	Pesticide exposure		Mann-Whitney U test	P-value
	Yes	No		
ALT	256.1	181.4	2894.5	0.001
AST	268.5	180.4	2548.5	0.001
ALP	228.9	183.6	3656.5	0.03

ALT, alanine aminotransferase; AST, aspartate aminotransferase; ALP, alkaline phosphatase.

P-values corrected for multiple comparisons using the Bonferroni method.

The association between environmental factors and AIH was assessed by examining the temperature zones in which participants resided. Among the AIH patients, 49.5% lived in the warm temperature zones, while 43% resided in moderate temperature zones, and only 7.5% lived in cold temperature zones. This distribution was significantly different from the controls, where only 20.4% lived in warm zones, with the remaining 79.6% in moderate or cold zones. The association between living in warm temperature zones and increased risk of AIH was statistically significant (X^2^ = 33.4, p-value 0.001), indicating that warm climates may contribute to the development of exacerbation of AIH in genetically predisposed individuals (**Tables 4**, **5**).

**Table 4:** The median and interquartile range of liver enzymes in Khat chewers.

Khat chewing			Liver enzymes		
			ALT	AST	ALP
Yes (N = 179)	Median		29	26	110
	Percentiles	25%	21	20	91
		50%	29	26	110
		75%	49	42	138
No (N = 194)	Median		24	24	103
	Percentiles	25%	16	19	82
		50%	24	24	103
		75%	33	30	126

**Table 5:** The median and interquartile range of liver enzymes in individuals with pesticides exposure.

Pesticides exposure			Liver enzymes		
			ALT	AST	ALP
Yes (N = 28)	Median		41	36	121
	Percentiles	25%	27	28	92
		50%	41	36	121
		75%	135	116	169
No (N = 345)	Median		26	24	104
	Percentiles	25%	17	19	85
		50%	26	24	104
		75%	37	32	129

Several factors were found to be associated with environmental exposure with AIH. Viral infections were notably among AIH patients, with 8.6% of patients reporting a history of past HBV. In terms of lifestyle factors, Khat Chewing was more common in AIH patients, with 65.6% of them reporting regular use compared to 42.1% of healthy controls (OR: 2.6, 95% CI: 1.6, 4.3, p-value <0.001). This suggests a strong environmental link between Khat chewing and the development of AIH (**Table 6**).

**Table 6:** The mean Vitamin D levels in association with AIH severity.

Vitamin D	AIH (N = 93)			One-way ANOVA	P-value
	Child-Pugh score				
	Class A N=62	Class B N=19	Class C N=12		
Mean ± SD	35.5 ± 2.88	24.1 ± 4.07	14.9 ± 4.63	227.3	0.001

P-values corrected for multiple comparisons using the Bonferroni method.

## 5 Discussion

This study aimed to explore the association between environmental triggers and autoimmune hepatitis in Yemeni patients. The findings indicate that AIH is influenced by a combination of environmental factors such as viral infections, toxins, medications, and dietary habits. Our results revealed that environmental factors, particularly Khat chewing and viral hepatitis, are significantly associated with the development of the severity of AIH in Yemen. A *post-hoc* sensitivity analysis excluding the 26 patients with prior exposure to nitrofurantoin or minocycline (27.9% of AIH group) yielded similar associations for Khat use and geographic zone, suggesting the findings are robust even when potentially drug-induced cases are removed.

The association between living in warm zones and increased AIH risk is an intriguing finding. Warm climates are often associated with various environmental stressors such as high exposure to UV radiation, temperature-related immune activation, and possibly heat-related immunosuppression. The number of participants reporting pesticide exposure was relatively small (n=8 among cases), which may limit statistical precision and widen confidence intervals. The data about history of pesticide exposure were not well identified and it were taken from patient itself. It is important to consider that warmer zones in Yemen are also regions of higher agricultural activity, which may lead to greater exposure to pesticides. This raises the possibility that the observed association between warm zones and AIH risk may, at least in part, reflect confounding by pesticide exposure rather than a direct effect of temperature. However, in our multivariate analysis, both warm zone residence and pesticide exposure remained independently associated with AIH, suggesting that while correlated, each may contribute distinctively to disease risk. Further spatial and occupational exposure mapping would be valuable in future studies to clarify this relationship.

To address concerns regarding reverse causation, we confirmed that Khat use preceded AIH diagnosis in all patients. Furthermore, cultural perceptions in Yemen do not associate Khat with medicinal use. In some cases, patients stopped chewing Khat following physician advice, further supporting its non-therapeutic role.

It is also important to consider that vitamin D deficiency in advanced AIH may represent a consequence of chronic liver dysfunction, given impaired hepatic hydroxylation and reduced nutritional status in cirrhotic patients. Therefore, while vitamin D may contribute to immune dysregulation, reverse causation cannot be excluded in advanced disease stages. Given the cross-sectional assessment of vitamin D levels, it is not possible to determine whether deficiency represents a causal factor or a consequence of advanced liver dysfunction.

Recent studies suggest that temperature fluctuations and heat stress can modulate immune response, potentially contributing to the development of autoimmune diseases (11). For example, heat stress can trigger the release of pro-inflammatory cytokines, which may exacerbate the immune response and contribute to the autoimmune process (12).

The interplay between environmental triggers and the development of AIH is still being documented. The studies have highlighted the role of viral infections as important Environmental factors in AIH pathogenesis. HBV, HCV, and EBV are all known to trigger immune responses in genetically predisposed individuals, leading to autoimmune liver damage (13, 19). The current study found that a significant proportion of AIH patients reported a history of these viral infections, supporting the hypothesis that viral hepatitis can exacerbate autoimmune responses and contribute to liver inflammation (14).

The association between AIH and HBV as a trigger related to environmental factors may delineate key factors surrounding AIH pathogenesis, and the specific role of environmental components is not fully understood, with further research being needed in this aspect.

All our patients were seronegative for anti-HCV antibodies, and no association between HCV infections and AIH was noted. Low prevalence of HCV (1.9%) infection was reported in our country (15), and the suggestive role of HCV as a trigger for AIH was reported in AIH-2, which was not identified in any of our patients. All our patients and the majority of healthy controls were positive for anti-HAV IgG, and this belongs to the increase prevalence of HAV infection in Yemen country as one of the developing countries which suffering from war, poverty, many socioeconomic statuses, and poor public health measures. Another study in Yemen reported that the seroprevalence of anti-HAV IgG reached 100% in adults (16).

In our study, the exclusion of past EBV infection as a trigger in our patients was also due to the increasing prevalence of anti-EBV IgG in Yemeni individuals, which reaches 82% in healthy people (17).

A previous study reported that the associations between HAV, HBV, and EBV infections were reported in AIH-1 patients, with no specific autoantigens identified; thus, no molecular mimicry could be established. However, specific markers for these viral infections are not found in the majority of AIH patients (18). Further studies should focus on the roles of HAV and EBV in AIH development.

Furthermore, the role of toxins in medications as environmental triggers was also evident. Pesticides and certain medications known to induce liver injury (e.g., Nitrofurantoin, Minocycline) were more common in AIH patients compared to controls. This aligns with previous research which was which has established that exposure to environmental chemicals and certain drugs can trigger autoimmune reactions instance, drugs like anti-TNF agents and statins are known to be associated with drug-induced autoimmune hepatitis (DIAIH) (20).

The significant association between Khat chewing and AIH in this study supports findings from previous research suggesting that Khat, a stimulant commonly used in Yemen, may play a role in immune system dysregulation (21). Studies have shown that Khat contains alkaloids, such as cathinone and cathine, that can alter immune function, potentially triggering autoimmune responses (22). These findings are consistent with a growing body of literature suggesting that Khat chewing is an important environmental factor contributing to AIH and other liver diseases.

The immune-modulating effects of Khat, including changes in cytokine production and immune cell activation, are likely to trigger the autoimmune process in susceptible individuals (23). Given that Khat chewing is prevalent among Yemeni males, it is plausible that the high incidence of AIH in this group is linked to their increased exposure to health and its effect on immune regulation (22). Although frequency of Khat chewing was assessed, precise quantification of cumulative exposure was limited. Dose–response relationships are important in strengthening causal inference, and future studies incorporating detailed exposure quantification would further clarify biological plausibility.

A study found that HBV was present in a significant proportion of AIH patients, and these findings are in line with those of research, which suggested that HBV infection plays a crucial role in triggering autoimmune hepatitis in generally predisposed individuals (24).

Another finding was the exposure to environmental toxins, such as pesticides, which were higher in AIH patients than in controls. Chemical exposure has been widely linked to the development of autoimmune diseases, including AIH (20). Previous studies have demonstrated that exposure to xenobiotics like carbon tetrachloride and trichloroethylene can induce immune dysfunction, leading to autoimmune responses in the liver (25). This study found a significant association between pesticide exposure and elevated liver enzyme levels, which further suggests that environmental pollutants may play a role in AIH development.

Certain medications, such as Nitrofurantoin, are known to cause drug-induced autoimmune hepatitis (26). The higher frequency of medication use among AIH patients in our study supports the findings of a researcher who suggested that drug-induced liver injury is an important environmental trigger in genetically susceptible individuals (27).

Our findings are consistent with current models of AIH pathogenesis that emphasize complex gene–environment interactions. Genetic predisposition, especially involving HLA-DR alleles, may interact with environmental triggers such as xenobiotics and viral infections to initiate autoimmune liver damage. Recent reviews underscore how epigenetic regulation, including histone modification and DNA methylation, may act as a bridge between environmental exposure and immune dysregulation (29, 30). These mechanisms may explain why only a subset of exposed individuals develop clinically evident disease, highlighting the multifactorial nature of AIH.

Vitamin D deficiency in advanced AIH may represent both a contributor to immune dysregulation and a consequence of chronic liver dysfunction. The liver plays a central role in vitamin D hydroxylation, and advanced fibrosis or cirrhosis may impair this metabolic pathway (31, 32). Additionally, malnutrition and reduced sunlight exposure in chronically ill patients may further contribute to deficiency. Therefore, reverse causality cannot be excluded.

Due to the high background seroprevalence of anti-HAV and anti-EBV IgG antibodies in the Yemeni population, seropositivity primarily reflects past exposure rather than acute or reactivated infection. In high-endemic regions, IgG-based serology lacks discriminatory capacity for causal inference (33). Therefore, we were unable to adequately assess the temporal relationship between these viral infections and AIH onset.

While this study provides valuable insights into the environmental triggers of AIH in Yemeni patients, there are several limitations to consider. The case-control design of this study limits our ability to establish a direct causal relationship between environmental triggers and the development of AIH. Longitudinal studies would be better in the future to understand the temporal relationship between these exposures and disease progression. This study relied on retrospective self-reporting of pesticide exposure, and no environmental or biological confirmation of specific agents was performed. Furthermore, recall bias and lack of precise timing relative to AIH onset limit our ability to infer causality. Future studies should employ prospective designs with biomonitoring or job-exposure matrices to better characterize chemical exposures. While age and sex matching were performed between cases and controls, the possibility of unmeasured confounders, like dietary habits, remains. While past exposure to HAV and EBV is nearly universal in the Yemeni population, the potential role of acute infection or viral reactivation as environmental triggers for AIH cannot be excluded. Our reliance on total IgG serostatus did not allow us to distinguish recent or reactivated infections, which may differ between cases and controls. This constitutes a limitation of the study, and future work should incorporate IgM serology or viral load assessments to explore this dimension more accurately.

The results of this study have significant implications for public health, particularly in regions where environmental exposures are prevalent. Even in the association between environmental exposures and AIH, public health efforts should focus on raising awareness about the potential risks of environmental triggers. In addition, early screening for viral infections and medication use in at-risk populations could help in the early diagnosis and prevention of AIH, while making the public aware of Khat chewing and pesticide exposure and their association with AIH.

## 6 Conclusion

This study demonstrates that environmental factors, particularly Khat chewing, pesticide exposure, viral infections, and residence in warm climate zones, are significantly associated with the development and severity of autoimmune hepatitis in Yemen. These exposures were linked to higher liver inflammation and more advanced fibrosis, emphasizing the role of environment-mediated immune dysregulation. The findings highlight the need for region-specific public health strategies, early screening, and clinical monitoring. Future studies should investigate the biological mechanisms underlying these associations to strengthen causal understanding.

## Data Availability

The original contributions presented in the study are included in the article/supplementary material. Further inquiries can be directed to the corresponding author.
